# Protein Ontology: a controlled structured network of protein entities

**DOI:** 10.1093/nar/gkt1173

**Published:** 2013-11-21

**Authors:** Darren A. Natale, Cecilia N. Arighi, Judith A. Blake, Carol J. Bult, Karen R. Christie, Julie Cowart, Peter D’Eustachio, Alexander D. Diehl, Harold J. Drabkin, Olivia Helfer, Hongzhan Huang, Anna Maria Masci, Jia Ren, Natalia V. Roberts, Karen Ross, Alan Ruttenberg, Veronica Shamovsky, Barry Smith, Meher Shruti Yerramalla, Jian Zhang, Aisha AlJanahi, Irem Çelen, Cynthia Gan, Mengxi Lv, Emily Schuster-Lezell, Cathy H. Wu

**Affiliations:** ^1^Protein Information Resource, Georgetown University Medical Center, WA 20007, USA, ^2^Center for Bioinformatics and Computational Biology, University of Delaware, Newark, DE 19711, USA, ^3^Department of Bioinformatics and Computational Biology, The Jackson Laboratory, Bar Harbor, ME 04609, USA, ^4^Department of Biochemistry and Molecular Pharmacology, NYU School of Medicine, NY 10016, USA, ^5^Department of Neurology, University at Buffalo School of Medicine and Biomedical Sciences, Buffalo, NY 14203, USA, ^6^Center of Excellence in Bioinformatics and Life Sciences, University at Buffalo, Buffalo, NY 14203, USA, ^7^Department of Immunology, Duke University Medical Center, Durham, NC 27705, USA and ^8^Department of Oral Diagnostic Sciences, University at Buffalo School of Dental Medicine, Buffalo, NY 14214, USA

## Abstract

The Protein Ontology (PRO; http://proconsortium.org) formally defines protein entities and explicitly represents their major forms and interrelations. Protein entities represented in PRO corresponding to single amino acid chains are categorized by level of specificity into family, gene, sequence and modification metaclasses, and there is a separate metaclass for protein complexes. All metaclasses also have organism-specific derivatives. PRO complements established sequence databases such as UniProtKB, and interoperates with other biomedical and biological ontologies such as the Gene Ontology (GO). PRO relates to UniProtKB in that PRO’s organism-specific classes of proteins encoded by a specific gene correspond to entities documented in UniProtKB entries. PRO relates to the GO in that PRO’s representations of organism-specific protein complexes are subclasses of the organism-agnostic protein complex terms in the GO Cellular Component Ontology. The past few years have seen growth and changes to the PRO, as well as new points of access to the data and new applications of PRO in immunology and proteomics. Here we describe some of these developments.

## INTRODUCTION

Scientific discourse is predicated on mutual understanding, verification of information and the inference of new connections. Ontologies facilitate such discourse by providing formal definitions of and disambiguation for entities of interest. In addition, placing entities in an ontological context facilitates the ability to make connections over short and long ranges within and between networks. It is toward these goals that the Open Biological and Biomedical Ontologies (OBO) Foundry was created (1). The Protein Ontology (PRO) is an OBO Foundry ontology that defines classes of proteins and explicitly indicates how these classes interrelate. The (intended) scope of PRO is the totality of naturally occurring protein entities. PRO has been used to define dendritic and hematopoietic cell types (2,3), to describe biological processes, to flag protein entities mentioned in the literature (4) and to capture information isolated from the literature in text mining workflows (5,6).

**Figure 1.**
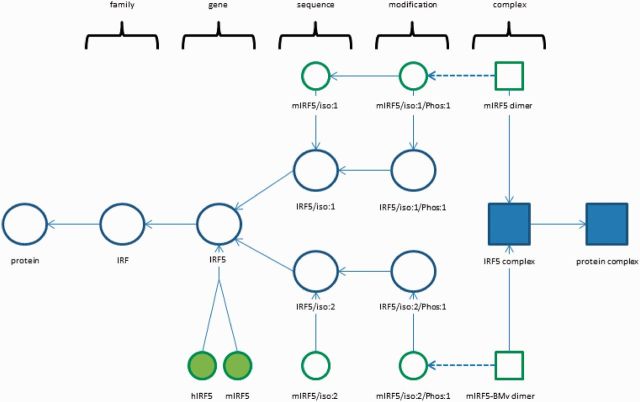
Illustration of PRO categories and relation to external resources. Categories (aka metaclasses) are listed along the top, with example terms for IRF5 (interferon regulatory factor 5) shown directly below. Circles denote protein terms, whereas squares denote protein complex terms. For each, blue indicates terms that are defined in an organism-agnostic way; green indicates terms that are organism-specific. Solid lines show is_a relationships and broken lines show has_component relationships (though the complexes in this example have components in the modification category, this need not be the case). Filled circles and filled squares indicate how UniProtKB and GO, respectively, fit into the hierarchy. Not all terms and relationships are shown. IRF, interferon regulatory factor; Phos, phosphorylated; m, mouse; h, human; iso, isoform; BMv, bone marrow variant.

Classes defined in PRO range in specificity from protein families to classes whose member proteins have specific post-translational modifications, are part of specific complexes, and which occur in specific organisms. The levels of specificity form the basis for categorizing PRO terms [(Figure 1; fully described in (7)]. Briefly, each PRO term categorized as ‘family-level’ refers to the class of proteins translated from a specific set of ancestrally related genes. A PRO term categorized as ‘gene-level’ refers to the class of proteins translated from a different gene related by 1:1 orthology in distinct organisms. Each term categorized as ‘sequence-level’ refers to the class of proteins translated from a specific mRNA of a given gene or from a specific translation start site. A term categorized as ‘modification-level’ refers to the class of all proteins modified in some specific way, either by amino acid modification or by proteolytic cleavage. And finally a term categorized as ‘complex’ refers to the class of complexes with a specific defined subunit composition. PRO makes no distinction between complexes whose components are modified before—or after—complex formation, and all complexes are grouped into the ‘complex’ category regardless of the specificity of their components.

As an OBO Foundry ontology, PRO is dedicated to interoperability not only with other ontologies but also with existing protein-related resources such as UniProtKB (8). For example, each organism-agnostic category described earlier in text also has organism-specific subcategories, so an ‘organism-gene’ PRO term would denote any translation product of a specific gene in a single organism. The latter, of course, are precisely what UniProtKB entries represent. Thus, PRO both uses UniProtKB and complements it by providing formal definitions for protein entities and placing the terms in an ontological context. PRO makes use of ontology resources such as PSI-MOD (9) and SO (10) to define protein classes of the modification category. PRO also makes use of community orthology sets with persistent accession numbers to provide the ability to group related proteins across the organisms represented in PRO. These sets are used to make connections between UniProtKB and PRO, with specific connections given in the form of a mapping between accessions from the two resources (7). In addition, the organism-specific protein complexes defined in PRO extend the (parental) hierarchy found in the Gene Ontology (GO) Cellular Component Ontology (11), the protein complex branch of which corresponds to PRO’s ‘complex’ category. The organism-agnostic complexes of GO thus provide parent terms for PRO’s organism-specific complex terms and provide the basis for connecting and comparing complexes between organisms.

The past few years have seen growth and changes to the ontology, as well as new points of access to the data and new applications. Here we describe some of these developments.

## ENHANCEMENTS OF DATA AND COVERAGE

### Reference organisms

Terms in the gene category comprise the largest metaclass within PRO. Terms in this metaclass are typically defined with respect to a gene in a reference organism, and the class of proteins covered by the term includes all translation products not only of that gene in that organism but also of any of its 1:1 orthologs in other organisms. Until 2012, when PRO defined terms using only human, mouse and *Escherichia coli* as references, users could reliably discern the reference organism from the gene name (the casing of which differs for each of the three organisms). For example, a protein defined as the translation product of the ADA gene (PR:000003707) meant the human ADA (adenosine deaminase) gene and its 1:1 orthologs, not the translation product of the *E. coli* ada (bifunctional transcriptional activator/DNA repair enzyme Ada) gene (PR:000022054) and its 1:1 orthologs. However, the addition of terms using other organisms as a reference (see later in text) has rendered this mechanism inadequate, especially since non-orthologous genes in different organisms can be given the same designation even in standard nomenclatures. For example, tubulin–tyrosine ligase (PR:000016788) is defined with respect to the human TTL gene, whereas uric acid degradation bifunctional protein TTL (PR:000035855) is defined with respect to the *Arabidopsis thaliana* TTL gene. In general, model organisms with well-established nomenclatures are used as references; preference is given to human, whereas *E. coli* is used for prokaryote-only classes, *Saccharomyces cerevisiae* for fungi-only classes and *A. thaliana* for plant-only classes.

### Mappings to external databases

We have improved, formalized and expanded the mapping of accessions from external databases to PRO identifiers. The majority of mapped accessions for protein records is from UniProtKB (http://www.uniprot.org), Reactome (http://www.reactome.org/) and EcoCyc (http://ecocyc.org/) databases. In addition, we provide gene record mappings from Mouse Genome Informatics (MGI; http://www.informatics.jax.org/), HUGO Gene Nomenclature Committee (HGNC; http://www.genenames.org/), EcoGene (http://ecogene.org/), PomBase (http://www.pombase.org/) and the Saccharomyces Genome Database (SGD; http://www.yeastgenome.org/). Previously these mappings were given only in a tab-delimited file that contains very limited information; now, however, they have also been formalized and expanded into a supplementary OBO-format file (promapping.obo, see later in text) that contains complete definitions of and relationships between the mapped terms. For example, we indicate that human follistatin protein (UniProtKB:P19883) is a subtype of follistatin protein found in any organism (PR:000000015) and is encoded by the human follistatin gene FST (HGNC:3971). The PRO mappings, which previously covered only three organisms (human, mouse and *E. coli*), now cover 12 GO reference proteomes (12): human, mouse, rat, zebrafish, chicken, fruit fly, budding yeast, fission yeast, *E. coli*, *A. thaliana*, *Caenorhabditis elegans* and *Dictyostelium discoideum*. Assignment of each organism-specific protein to a PRO organism-agnostic gene category term is done (largely) automatically using 1:1 ortholog inferences provided by OMA (13). Mapping statistics for each release are provided at the PRO website.

### Reuse of UniProtKB accessions

PRO reuses external ontology identifiers whenever feasible. Thus, if a required term is already defined in another ontology, PRO uses that term without changing its identifier (i.e. we do not create an identical term and assign a PRO identifier to it). However, much information in the protein field is available in the UniProtKB database, with established and stable accessions. Therefore, when we incorporate UniProtKB entries into the PRO ‘organism-gene’ category (Figure 1, filled green circles), in the interest of minimizing the minting of new identifiers, we use UniProtKB accession identifiers directly, prefixing them with ‘PR:’ as in the example PR:P19883 (‘http://purl.obolibrary.org/obo/PR_P19883’ for the URL). All such entries are cross-referenced back to the originating database, using ‘UniProtKB:’ as the prefix, as in the example ‘UniProtKB:P19883’ (‘http://www.uniprot.org/uniprot/P19883’ for the URL). In this way, the database and ontological representations of P19883 are clearly distinguished. In the future, use of UniProtKB isoform identifiers (of the form P19883-1) will be adopted.

## NEW COLLABORATIONS AND AREAS OF APPLICATION

### ImmPort

PRO Consortium scientists are working with scientists at Stanford within the framework of the Bioinformatics Integration Support Contract (BISC) and the Human Immunology Project Consortium (HIPC; http://www.immuneprofiling.org/hipc/page/show). The goal of BISC is to collect and house data in the Immunology and Data Analysis Portal (ImmPort) and to make these data accessible on the web. An important part of ImmPort’s data and of the associated work by HIPC scientists derives from the application of the innovative CyTOF technique, a variation of mass cytometry in which antibodies are labeled with heavy metal ion tags rather than with fluorochromes to allow single samples to be probed simultaneously with many antibodies (14). This, however, creates challenges for cell typing, as for many cell populations the CyTOF-generated data go far beyond our current knowledge.

The Cell Ontology (15) defines cell types. To enhance the utility of the CL to support automated analyses of cell types, an enhanced association of markers to particular cell types is needed, so that each cell type is associated with all markers it is known to express. A problem arises here because immunologists typically use multiple different names for the proteins identified in their experiments. PRO is thus used as controlled vocabulary to yield consistent data for analysis while at the same time providing complete lists of synonyms associated with proteins of interest to allow for proper mapping of the data (Table 1). In addition, because practitioners of flow cytometry and CyTOF use antibodies as primary reagents to detect cellular markers, an adequate analysis and integration of their data requires a comprehensive mapping of antibodies to the proteins they detect. We are curating a mapping between such antibodies and PRO IDs, thereby indicating the specific modifications that might be targeted in experiments and reference to the appropriate level of specificity. For example, anti-CD45 monoclonal antibody clone HI30 recognizes all isoforms of the CD45 marker, whereas HI100 recognizes only the CD45RA isoform (Table 1). Src provides another example. Anti-Src clone 3828 recognizes only human Src of all forms, whereas K98-37 recognizes only the form phosphorylated (at least) on tyrosine-418 (human numbering); it is, however, predicted to cross-react with the corresponding form in other species.

**Table 1.** Portion of antibody registry

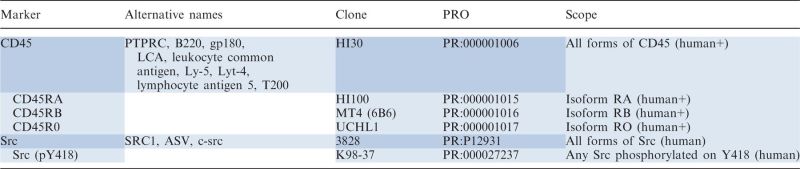

Shading in the marker column indicates that the named marker includes the shown forms. For example, CD45RA is one isoform of CD45. Shading in the clone column indicates that the clone recognizes the shown subtypes. Shading in the PRO column indicates that the shown classes are subtypes. The Scope column indicates what forms are included in the PRO class. Not all alternative names and clones are shown.

### Proteoform repository

The PRO Consortium is collaborating with the top-down proteomics community from Northwestern University (http://www.topdownproteomics.org/). The top-down proteomics approach focuses on intact protein molecules, rather than their pieces. Thus, results more accurately reflect the structures and properties of proteins in actual biological systems than do results using a bottom-up proteomics approach (16) and allow the identification of multiple simultaneous modifications. The Proteoform Repository is a database that contains experimentally verified proteoforms—isoforms and post-translationally modified forms—from various cell types in three model organisms (human, mouse and yeast) (17). PRO provides the corresponding ontological representations with links to this data source (e.g. PR:000036126). The formal representation of proteoforms in PRO will enable (i) the study of conserved modifications among these organisms, (ii) the association to functional information and (iii) uncovering relations between proteoforms. In addition, inclusion of the metadata related to the cell type(s) where the proteoforms were identified will allow comparison among different cell types or in normal versus disease states. PRO Release 37 contains 194 proteoforms encoded by 164 genes. Eighteen were found to be conserved between human and mouse. Considering only the highest confidence subset of the current repository data, we expect to integrate >700 proteoforms, ∼400 of which contain multiple modifications.

### Representations of biological pathways using PRO

PRO provides unique identifiers for each form of proteins and protein complexes, and thus it is a useful resource for those seeking to represent pathways—particularly signaling and regulatory pathways—which often involve changes in post-translational modification of proteins and differences in composition of complexes. Moreover, the organization of protein forms by orthology in PRO enables cross-species comparisons (6,18). Recently, PRO has been used to describe two such pathways: Toll-like receptor signaling and the spindle checkpoint.

TLRs are transmembrane receptors that facilitate recognition of microbial molecules. Activation of the receptors induces an intricate chain of protein–protein interactions regulated by the recruitment of different intracellular adaptors. Furthermore, the pathways are not linear; they can diverge or merge to produce synergistic responses that are not predictable from the output evoked by an individual canonical pathway. The spindle checkpoint maintains genomic integrity during mitosis and meiosis by inhibiting chromosome segregation until all chromosomes have been correctly attached to the spindle. Checkpoint signaling involves numerous protein phosphorylation events. One key checkpoint protein, BUB1B, is phosphorylated on at least 12 different sites by three different protein kinases. Four phosphorylated forms of BUB1B are defined in PRO, each phosphorylated on a different biologically relevant subset of the 12 sites. Each form is functionally annotated to describe its characteristic protein–protein interactions and role in the spindle checkpoint and related processes.

### Ambiguous protein forms

PRO can represent both intentional ambiguity (e.g. a complex can include any one of a family of proteins (19) and genuine uncertainty (e.g. a protein is known to be phosphorylated but the identity of the modified residue is unknown) for protein forms. The ability to do so has practical applications in scientific discourse, as it is sometimes necessary to refer to collections of objects made either for the sake of convenience or to express incomplete knowledge. One example of this is osteocalcin peptide, which exists in three forms that are distinguished by the carboxylation state of three specific glutamic acid (glu) residues (which can be converted to gamma-carboxyglutamic acid, gla) (20,21). Fully carboxylated osteocalcin contains three gla residues, the undercarboxylated form contains one or two and the uncarboxylated form contains none. Assays can distinguish between the three, but cannot distinguish between the possible undercarboxylated forms. PRO thus contains a general term for undercarboxylated osteocalcin (PR:000024926), defined as containing a minimum of one gla residue and a maximum of two, without indicating the positions of modification.

## NEW WEB VIEWS AND RESOURCES

### Organism–gene summary view

**Figure 2.**
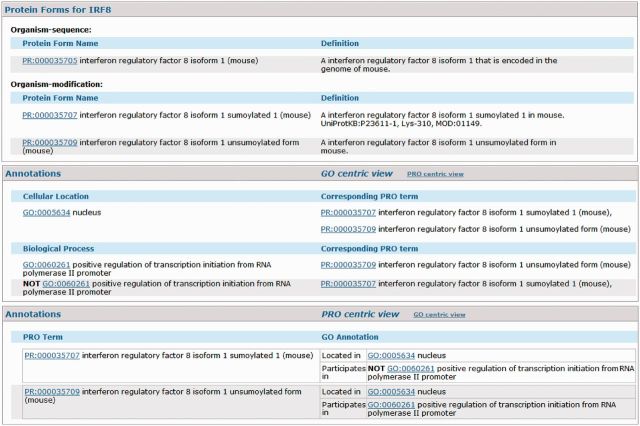
Display for protein products of the mouse Irf8 gene. Top panel, defined PRO terms; Middle panel, GO-centric view of annotations; Bottom panel, PRO-centric view of the annotations.

Annotation efforts are focusing more and more on capturing information at specific levels. For example, the GO Consortium has modified its policies and data files so curators can indicate the precise form of a gene product for which an indicated annotation applies, be it a specific isoform or a post-translationally modified form. Accordingly, we have enhanced the web view for terms in the organism–gene metaclass. Whereas the web view previously displayed information pertaining solely to the displayed term, the new view presents all the products of a specific gene (corresponding, in most cases, to a single UniProtKB entry) and presents all the annotation associated with each product (Figure 2). Scientists can thus compare these different products directly, either using the GO-centric view (middle panel), where the relevant products of the gene are mapped to each GO annotation, or the PRO-centric view (bottom panel), where the relevant GO annotations are shown for each gene product. For example, mouse interferon regulatory factor 8, encoded by the Irf8 gene (MGI:96395; protein sequence given in UniProtKB: P23611), can be sumoylated. When in the unsumoylated form (PR:000035709), the protein can positively regulate RNA polymerase II transcription initiation (GO:0060261). The sumoylated form (PR:000035707), however, cannot. This difference is made clear by the new view. The approach described here for GO will be extended to other ontologies that are used for describing other attributes of PRO terms, such as involvement in disease, and absence or presence of protein domains.

### Cytoscape view

**Figure 3.**
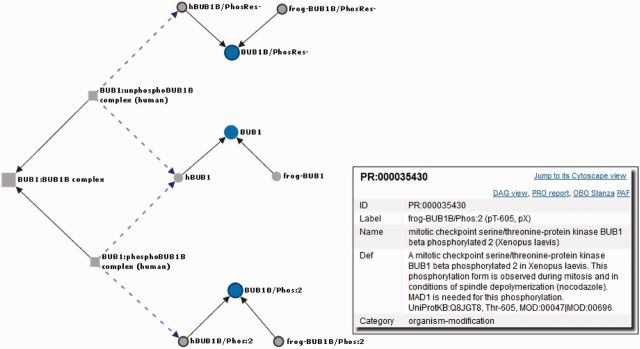
Cytoscape view of BUB1:BUB1B complexes. The is_a relationship between child and parent in the PRO hierarchy and the has_component relationship between complexes and their components are shown by black arrows and blue dotted arrows, respectively. Clicking on a node (e.g. frog-BUB1B/Phos:2) brings a pop-up box that provides details and allows additional actions. Only a subset of nodes is shown, which have been rearranged for clarity.

PRO now features a Cytoscape view that provides an interactive and visual interpretation of PRO terms and their relations. The view can be accessed via the Cytoscape icon displayed on PRO search results pages and entry reports. Cytoscape supports searches and customization of the layout and display; displays links to PRO entry pages, OBO stanzas and annotations; and allows saving of the network figure in PNG format. Networks pertinent to a single entry or to multiple entries selected from search results pages can be shown. The default view displays the hierarchical relationships for parents, siblings and children of the requested entries. For complexes, the hierarchies for both the complexes and the complex components are displayed. For example, Figure 3 shows the Cytoscape view for complexes containing the kinase, BUB1 and a related kinase BUB1 beta (aka BUBR1) in either the unphosphorylated form (upper part) or one possible phosphorylated form (lower part). The figure illustrates how analogous complexes in another organism can be inferred based on the displayed network. For example, one can follow has_component relationships for the unphosphorylated version of the human complex to see that each of its components—human BUB1 and human unphosphorylated BUB1 beta—has a counterpart in *Xenopus laevis* [follow the is_a relationship to the organism-agnostic form (blue circles), then back down the other branch to the *Xenopus* version]. The conservation of components could indicate that *Xenopus* too has such a complex, though currently experimental evidence is lacking.

## ACCESS TO PRO

PRO is accessible via the Internet at http://proconsortium.org. The home page includes a brief overview of the ontology and links to search and browse functions, statistics, documentation/tutorials, term request and annotation pages and downloads.

Downloadable files include the main PRO OBO file (pro.obo), a supplementary OBO file containing terms for mapped entries from external databases (promapping.obo), the PRO annotation file (PAF.txt) and a tab-delimited mapping file (promapping.txt). In addition to the two OBO files indicated earlier in text, versions of the OBO files to which logical reasoning has been applied are available (pro_reasoned.obo and promapping_reasoned.obo). The reasoned files, produced using the ELK reasoner of the OBO Ontologies Release Tool (http://code.google.com/p/owltools/wiki/OortIntro), contain additional inferred relations that, for brevity and simplicity, are not explicit in the non-reasoned versions. Before reasoning, all PRO terms are defined using a single-parent hierarchy; reasoning in some cases adds new parent terms.

Links to PRO terms can be made using the general form http://purl.obolibrary.org/obo/PR_xxx, where xxx denotes either a nine-character numeric string for the traditional PRO identifier or a six-character alphanumeric string indicating a UniProtKB accession. UniProtKB-derived PRO terms are cross-referenced from within the corresponding UniProtKB entries.

Users can request PRO terms or contribute to the curation of PRO terms. The PRO Tracker (Sourceforge) is the main mechanism for requesting new terms or modification of existing ones. RACE-PRO (22) is a user-friendly interface that allows the addition of experimental information to PRO terms, and therefore fosters contribution by domain experts.

## FUNDING

National Institutes of Health (NIH) [5R01GM080646-08]; and the National Science Foundation [DBI-1062520]. Additional support was provided by the National Instititutes for Allergy and Infectious Diseases [contract HHSN272201200028C to A.D.D., A.R. and B.S.]. Funding for open access charge: NIH [5R01GM080646-08].

*Conflict of interest statement*. None declared.
